# Tooth Powders

**Published:** 1857-12

**Authors:** 


### DENTIFRICES

OUGHT to be stuffed down the throats of the gentlemen of the Plug Craft. The best Dentifrice in the world is rain water or soap-suds; all powders are absolutely injurious; and it is useless to enter into any argument on the subject. There is not a tooth-powder named, but, if rubbed on an iron bar with a tooth-brush, would not wear that bar in two. Dentists of renown tell us that the enamel, the covering of the tooth, is not renewable, does not grow, then set us about scrubbing it away with powders!

The next tom-foolery which has engaged their attention in the prim city of Boston, the Athens of the Western Hemisphere, in solemn conclave assembled, within a few months, is the influence which certain articles have in destroying teeth. They have issued a scientific bull, to the effect that the *main* cause of defective teeth is the use of *saleratus* and *cream of tartar*, in the manufacture of bread: the proof being, that if a tooth is put in a strong solution of saleratus it will be dissolved! Molasses will do the same thing.

There are two questions which present themselves just here: How can it be managed to soak a tooth a week or two in strong saleratus water? It seems to us they would have to pull the tooth out; then there would be no use in soaking it in anything. It would be better to throw it away, and save the trouble of a soak. It will rot in the ground, if left there long enough. Come to think of it, the Dental Society of New England would itself be dissolved by a soak, by any kind of soak, saleratus or brandy—'specially the brandy. But a tooth in a man's head is a *living thing,* and powerfully resists all destructive agencies; out of a man's head, it is dead, and is passive to any cause of decay. We cannot reason from life to death; it will always lead us to errors in practice and in theory.

But there is another inquiry which this most learned body failed to make. Does any body eat saleratus or cream of tartar? We venture the assertion, that not a grain of saleratus can be found in any slice of bread in all America, if that bread has been made with a small modicum of common sense.

Ordinarily, a level teaspoon of saleratus or cream of tartar is put in several pounds of flour; but that saleratus is put in with the express purpose of its decomposition: it would not have the effect designed, unless it was decomposed; and when thus decomposed, it is no longer saleratus, no longer an alkali which would dissolve a tooth, but it has changed its whole nature, and become glauber salts, which is one of our most harmless articles of household medicine. A tablespoon or two of glauber salt is an ordinary dose, and several hours after taken, it acts on the bowels; but in the ordinary eating of bread, it would take about six months for a person to swallow an amount of the salts to make a common dose of the glauber. This is Homœopathy.

We do not deny that it is a useless, foolish, and hurtful practice, to mix any saleratus in our food; it was the "invention" of some lazy lout; and no family ought to allow of its employment in any shape or form, except as a medicine, prescribed by a physician. But our objection is, that a body of scientific men should have allowed themselves to have been led by the nose by some monomaniac, of good intentions but of green wit: green as grass, verdant as—we don't know what.

Our teeth are decayed by dirty mouths, hot drinks, and foul stomachs. Wash the teeth well after each meal, in soft water, eat and drink nothing warmer than the blood—that is about a hundred degrees of Fahrenheit—let that eating be at regular hours, using in moderation plain, substantial food, with a plentiful amount of the perfect and ripe fruits of the earth; and thus set all ignorant and dishonest dentists to breaking rocks for the turnpike. Be assured, reader, that the best dentifrices in the world are: soft water, clean mouths, healthy stomachs, and moderate activities in the open air.

